# Self-assembled nano-PROTACs potentiate colorectal cancer immunotherapy via downregulating PD-L1 and GPX4 expression

**DOI:** 10.1016/j.mtbio.2026.103491

**Published:** 2026-07-23

**Authors:** Xueping Luo, Yixin Liu, Guangmiao Chen, Xinrui Ma, Pingping Xu, Yutong Li, Longyu Zheng, Rongrong Zheng, Youqin Xu, Shiying Li, Linping Zhao

**Affiliations:** a Key Laboratory of Biological Targeting Diagnosis, Therapy and Rehabilitation of Guangdong Higher Education Institutes, The NMPA and State Key Laboratory of Respiratory Disease, The Fifth Affiliated Hospital, Guangzhou Medical University, Guangzhou, 510700, PR China; b Guangdong Provincial Key Laboratory of Molecular Target & Clinical Pharmacology, The NMPA and State Key Laboratory of Respiratory Disease, School of Pharmaceutical Sciences and the Fifth Affiliated Hospital, Guangzhou Medical University, Guangzhou, 511436, PR China

**Keywords:** Self-assembly, PROTAC, Ferroptosis, Immunogenic cell death, Immunotherapy

## Abstract

Ferroptosis, a form of regulated cell death that is iron-dependent and driven by the accumulation of lipid peroxides (LPO), has emerged as a promising avenue for enhancing tumor immunogenicity and augmenting immune checkpoint blockade (ICB) efficacy. In this study, we demonstrated that dBET57, a BRD4-targeting PROTAC, potently suppresses the expression of GPX4, the master negative regulator of ferroptosis, thereby triggering ferroptotic cell death, while concurrently downregulating PD-L1 to reverse immune evasion. Based on this, we developed a self-assembled nano-PROTAC platform, termed dBET@TF, designed to amplify ferroptosis and potentiate colorectal cancer immunotherapy. Specifically, dBET@TF was constructed via self-assembly of dBET57, tannic acid, and Fe^3+^, which endowed the formulation with acid-responsive drug release capability and significantly improved its intracellular delivery efficiency. Mechanistically, dBET@TF promoted ferroptosis through iron accumulation and GPX4 depletion, triggering robust immunogenic cell death (ICD). Concurrently, BRD4 degradation mediated by dBET@TF durably suppressed PD-L1 expression, reprogramming the tumor microenvironment to foster enhanced immune activation, characterized by increased infiltration of natural killer (NK) cells and cytotoxic T lymphocytes (CTLs). As a result, dBET@TF significantly potentiated the therapeutic response to αPD-1 in both primary tumor and experimental lung metastasis models. Overall, this work establishes a novel PROTAC-based therapeutic strategy that leverages BRD4 degradation to amplify ferroptosis and remodel antitumor immunity, offering a promising approach to potentiate colorectal cancer immunotherapy.

## Graphical abstract

Self-assembled nano-PROTACs (designated as dBET@TF) were fabricated through the co-assembly of dBET57, tannic acid and Fe^3+^. dBET@TF synergistically amplified ferroptosis by elevating intracellular iron levels and downregulating GPX4, thereby inducing robust immunogenic cell death. Concurrently, BRD4 degradation by dBET@TF led to reduced PD-L1 expression, eliciting a markedly enhanced immune response, which significantly improved the efficacy of ICB therapy in both primary tumor and experimental lung metastasis models.

**Figure:**
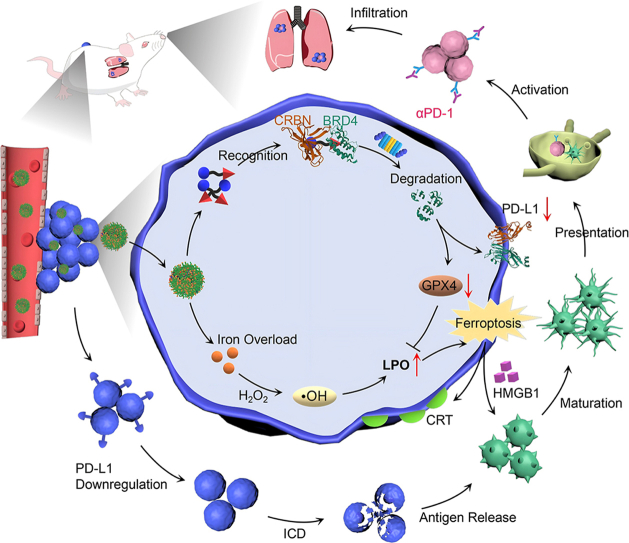

## 1 Introduction

**Scheme 1:**
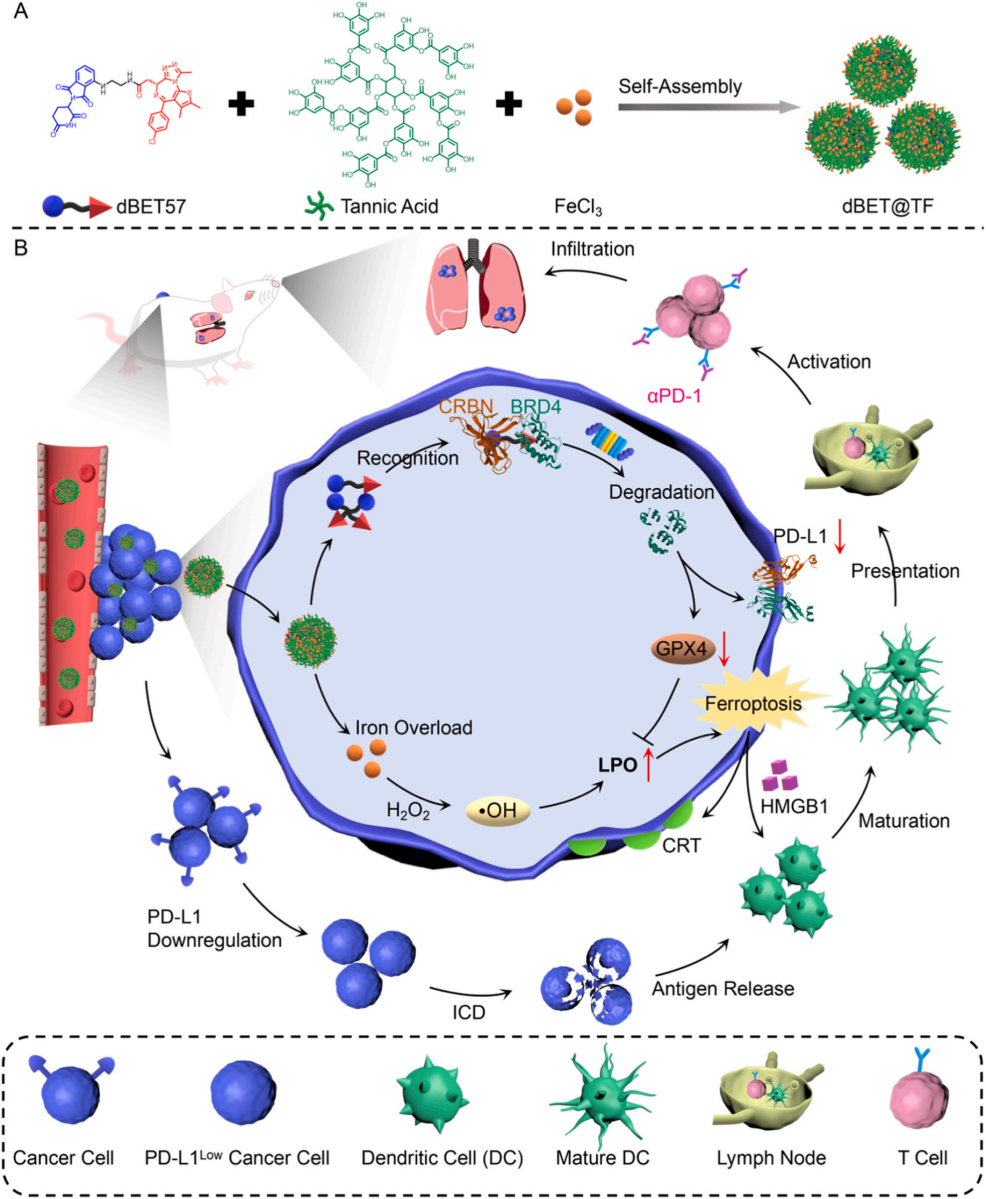
Schematic drawing of dBET@TF to amplify antitumor immunity via dual ferroptosis induction and PD-L1 suppression. (A) dBET@TF was fabricated via the self-assembly of dBET57, tannic acid, and Fe^3+^, forming a stable, iron-rich nanostructure with inherent ferroptosis-promoting capacity. (B) Upon systemic administration, dBET@TF preferentially accumulated at the tumor site via passive targeting. Once internalized, dBET@TF synergistically triggered ferroptosis by elevating intracellular iron and downregulating GPX4, thereby inducing immunogenic cell death. Concurrently, BRD4 degradation by dBET@TF led to reduced PD-L1 expression, eliciting a markedly enhanced immune response characterized by increased infiltration of natural killer cells and cytotoxic T lymphocytes. Ultimately, dBET@TF potentiated the antitumor efficacy of ICB therapy against primary tumor and experimental lung metastasis models.

Colorectal cancer remains one of the most commonly diagnosed malignancies worldwide, contributing substantially to cancer-related mortality [1,2]. Its high propensity for metastasis poses persistent clinical challenges, underscoring the urgent need for effective therapeutic strategies with durable benefits [3,4]. Over the past decade, immune checkpoint blockade (ICB), particularly with antibodies targeting programmed death-1 (PD-1), has fundamentally altered the therapeutic landscape for cancer by reactivating cytotoxic T lymphocytes [[5], [6], [7]]. However, the clinical benefit of αPD-1 therapy in colorectal cancer remains suboptimal, with limited response rates. A key impediment is the intrinsic heterogeneity and weak immunogenicity that characterize most colorectal tumors, in which impaired antigen presentation curtails effective T-cell infiltration and ultimately suppresses tumor-specific immune effector functions [[8], [9], [10]]. Accordingly, strategies that amplify tumor immunogenicity and render tumors sensitive to immune-mediated attack are urgently required to boost the efficacy of αPD-1 therapy (see Scheme 1).

Combining αPD-1 with complementary therapeutic modalities is increasingly recognized as a strategic avenue to surmount the persistent limitations of checkpoint blockade therapy [[11], [12], [13]]. In particular, approaches that trigger immunogenic cell death (ICD), a regulated form of cytotoxicity that liberates tumor-associated antigens together with danger signals, hold considerable promise for re-educating immunologically “cold” tumors into “hot” ones [[14], [15], [16]]. Within this therapeutic paradigm, ferroptosis, a non-apoptotic cell death pathway orchestrated by iron-mediated lipid peroxidation, has garnered significant attention. Ferroptosis not only leads to tumor cell elimination but also promotes the release of damage-associated molecular patterns (DAMPs), which recruits dendritic cells, prompting their maturation and, in turn, amplifying T-cell-mediated antitumor immunity [[17], [18], [19], [20]]. However, the intrinsic immunogenic capacity of ferroptosis is often suppressed by cellular defensive mechanisms. Specifically, glutathione peroxidase 4 (GPX4), the master negative regulator of ferroptosis, detoxifies lipid hydroperoxides and thereby confers resistance to ferroptotic cell death [[21], [22], [23]]. Concurrently, tumor cells frequently upregulate programmed cell death ligand 1 (PD-L1), which engages PD-1 receptors on activated cytotoxic T lymphocytes. This interaction inhibits T cell receptor signaling and imposes an immunosuppressive barrier, ultimately diminishing the pro-immune activation effects elicited by ferroptosis [[24], [25], [26]]. Of particular note, elevated expression of bromodomain-containing protein 4 (BRD4), an epigenetic reader and transcriptional co-regulator, has been implicated in the transcriptional activation of both GPX4 and PD-L1. Specifically, through its distinctive four-alpha-helix bundle structure, BRD4 recognizes acetylated histones and recruits transcriptional co-regulators to super-enhancer regions, thereby driving the expression of ferroptosis-related genes, including GPX4 [27,28]. In parallel, Several studies have demonstrated that BRD4 directly occupies the CD274 (PD-L1) gene promoter, and this binding is further enhanced upon IFN-γ stimulation [29,30]. These findings collectively position BRD4 as a critical upstream node that coordinately governs ferroptosis susceptibility and immune evasion in cancer cells. Thus, strategies targeting BRD4 are critical to unleashing the full immunogenic potential of ferroptosis and thereby enhancing the durability of ICB therapy in colorectal cancer.

While several BRD4 inhibitors, including JQ1, I-BET151 and OTX015, have exhibited encouraging antitumor activity in early-phase clinical studies, their clinical value is often compromised by an adaptive, feedback-driven upregulation of BRD4 protein, which in turn promotes resistance development within tumor cells, ultimately resulting in suboptimal anti-proliferative effects [31,32]. In contrast to conventional inhibitors that merely antagonize target function, proteolysis targeting chimeras (PROTACs) leverage the intracellular degradative machinery by actively recruiting the ubiquitin-proteasome system to induce targeted degradation of the protein of interest [[33], [34], [35]]. This degradation-based approach holds potential for enhanced sensitivity, improved selectivity, greater potency, and more dynamic control over protein function [36,37]. Despite these theoretical benefits, the clinical application of PROTACs remains constrained by significant pharmacokinetic drawbacks, including poor systemic stability, unfavorable metabolic profiles, and inefficient intracellular delivery [38,39]. Therefore, expediting the design of PROTAC-enabled nanoplatforms is essential to fully harness their synergistic therapeutic promise for colorectal cancer immunotherapy.

In this study, the BRD4-targeting PROTAC (dBET57) was shown to inhibit the expression of GPX4 and PD-L1, thereby inducing ferroptosis and reversing immune evasion. To enhance therapeutic performance and overcome delivery challenges, we developed self-assembled nano-PROTACs, termed dBET@TF, designed to amplify ferroptosis and potentiate colorectal cancer immunotherapy through simultaneous downregulation of PD-L1 and GPX4. Specifically, dBET@TF was constructed via self-assembly of dBET57, tannic acid, and Fe^3+^, which endowed the formulation with acid-responsive drug release capability and significantly improved its solubility and intracellular delivery efficiency. Mechanistically, dBET@TF synergistically amplified ferroptosis by increasing intracellular iron levels and downregulating GPX4, thereby inducing robust immunogenic cell death. Concurrently, BRD4 degradation by dBET@TF led to reduced PD-L1 expression, eliciting a markedly enhanced immune response characterized by increased infiltration of natural killer cells and cytotoxic T lymphocytes. Consequently, dBET@TF markedly potentiated αPD-1 therapy, yielding superior therapeutic outcomes in both primary tumor and experimental lung metastasis models, while also establishing protective immunological memory against tumor rechallenge. Collectively, this study demonstrates the promise of PROTAC-driven combinatorial regimens as a potent immunostimulatory platform capable of mounting antitumor immunity and enhancing the response to ICB in colorectal cancer.

## 2 Materials and methods

### 2.1 Materials

dBET57, a PROTAC used in this study, was purchased from MedChemExpress (USA). Iron(III) chloride hexahydrate (FeCl_3_·6H_2_O) and tannic acid were obtained from Aladdin (China). Cell viability and membrane integrity were evaluated using the Yeasen Live/Dead Cell Detection Kit (China). Lipid peroxidation was quantified with the C11-BODIPY probe (ThermoFisher, USA). MTT reagent (Biyuntian, China) was used to assess cytotoxicity of therapeutic agents. Western blot analysis was performed with primary antibodies against GAPDH, GPX4, PD-L1, calreticulin, and HMGB1, all of which were obtained from Abcam (UK).

### 2.2 Cell culture

Mouse colorectal cancer (CT26) cells were cultured in Dulbecco's modified Eagle's medium (DMEM) enriched with 10 % fetal bovine serum and 1 % antibiotic solution, then incubated at 37 °C in a humidified atmosphere bearing 5 % CO_2_.

### 2.3 Preparation of dBET@TF

To prepare dBET@TF, 35 μL of dBET57 stock solution (10 mg/mL in DMSO) and 15 μL of tannic acid stock solution (40 mg/mL in DMSO) were added dropwise to 1 mL of ultrapure water. Subsequently, 15 μL of FeCl_3_·6H_2_O stock solution (10 mg/mL in water) was injected into the mixture. The resulting solution was subjected to ultrasonic agitation for 10 min to promote formation. Subsequently, the suspension was centrifuged at 12 000 rpm for 30 min, yielding a solid precipitate. This precipitate was washed twice with ultrapure water to remove residual reagents and solvents, and the final product, dBET@TF, was collected for further studies.

### 2.4 Characterization of dBET@TF

Transmission electron microscopy (TEM) was used to visualize the morphology of dBET@TF, while dynamic light scattering (DLS) was used to measure its hydrodynamic dimensions and polydispersity index (PDI). The iron content of dBET@TF was quantified by inductively coupled plasma mass spectrometry (ICP-MS) using a standard calibration curve. Moreover, encapsulation efficiency was determined by high-performance liquid chromatography (HPLC). Briefly, dBET@TF was dissolved in DMSO and subjected to HPLC analysis. The mobile phase comprised a mixture of acetonitrile (mobile phase A) and 0.2 % trifluoroacetic acid (TFA) in water (mobile phase B) at a 60:40 (v/v) ratio. The encapsulation efficiency was calculated as follows: encapsulation efficiency (%) = (weight of encapsulated drug/total weight of drug used) × 100 %.

### 2.5 Cytotoxicity assay

CT26 cells (5 × 10^3^ cells/well) were seeded in 96-well plates and incubated at 37 °C to allow attachment. The cells were then treated with graded concentrations of TA/Fe, dBET57, or dBET@TF and incubated for 24 h. After drug exposure, MTT solution (prepared in PBS) was added to each well, and the plates were incubated in dark environment for 4 h. Subsequently, the solution was then carefully aspirated and DMSO was added to dissolve the formazan crystals completely. After that, absorbance of each well was measured at 570 nm using a microplate reader.

### 2.6 Cellular uptake analysis

Cellular uptake of dBET@TF was assessed using Cy5.5-labeled formulations. CT26 cells seeded on confocal dishes were exposed to fresh medium containing a range of Cy5.5-labeled dBET@TF concentrations for 24 h, or to the formulation for temporal analyses (3, 6, 9, and 12 h). After staining with DAPI, cells were visualized by confocal laser scanning microscopy (CLSM).

To elucidate the cellular uptake pathway of dBET@TF, CT26 cells were pretreated with Methyl-β-cyclodextrin (M-β-CD) (100 mM), 5-(N-ethyl-N-isopropyl) amiloride (EIPA) (50 mM) or pistop2 (10 mM) for 2 h. After that, the cells were incubated with fresh medium containing Cy5.5-labeled dBET@TF for an additional 6 h. Finally, the cells were stained with DAPI and visualized by CLSM.

### 2.7 Immunofluorescence analysis

CT26 cells were plated on confocal dishes and permitted to adhere for 12 h. After 24 h of treatment with the indicated agents, the cells were fixed in 4 % paraformaldehyde for 20 min, permeabilized with 0.1 % Triton X-100 for 10 min, and then blocked in PBS supplemented with 10 % goat serum for 1 h. Primary staining was performed with anti-CRT or anti-HMGB1 antibodies (overnight, 4 °C), followed by incubation with the corresponding fluorescent secondary antibodies. At last, fluorescence images were acquired on a CLSM.

### 2.8 Measurement of reactive oxygen species (ROS) production

CT26 cells were treated with TA/Fe, dBET57 or dBET@TF (with identical doses of 25 mg/L for dBET57 and 3.1 mg/L for Fe^3+^) for 24 h. Subsequently, cells were treated with the ROS probe DCFH-DA for an additional 40 min. The green fluorescence was visualized under CLSM. For quantitative analysis, cells treated as described above were harvested, incubated with DCFH-DA for 40 min, and analyzed by flow cytometry.

### 2.9 Detection of lipid peroxidation

After incubating with TA/Fe, dBET57 or dBET@TF (with identical doses of 25 mg/L for dBET57 and 3.1 mg/L for Fe^3+^) for 24 h, CT26 cells were incubated with C11-BODIPY (5 μM) for 30 min. Then, the fluorescence generated inside the cells was observed under CLSM and quantified.

### 2.10 Detection of extracellular ATP content

CT26 cells were treated with TA/Fe, dBET57 or dBET@TF (with identical doses of 25 mg/L for dBET57 and 3.1 mg/L for Fe^3+^) for 24 h. After that, the culture medium was collected and centrifuged to remove cell debris. A 50 μL aliquot of the supernatant was then mixed with the ATP detection working solution, and the ATP content was determined by measuring chemiluminescence using a luminometer.

### 2.11 Live/dead cell staining

CT26 cells were seeded onto confocal dishes and incubated 12 h to allow attachment. The adherent cells were then exposed to TA/Fe, dBET57 or dBET@TF (with identical doses of 25 mg/L for dBET57 and 3.1 mg/L for Fe^3+^) for 24 h. After staining with calcein-AM and propidium iodide (PI) for 30 min, the cells were visualized by CLSM.

### 2.12 dEBT@TF induced DCs maturation in vitro

Bone marrow-derived dendritic cells (BMDCs) were isolated from BALB/c mice and cultured in fresh medium supplemented with GM-CSF and IL-4. In parallel, CT26 cells were treated with PBS, TA/Fe, dBET57 or dBET@TF (at equivalent doses of 25 mg/L for dBET57 and 3.1 mg/L for Fe^3+^) for 24 h. Subsequently, the pretreated CT26 cells were co-cultured with the obtained BMDCs for an additional 24 h. BMDCs were then harvested and stained with antibodies against CD11c, CD80, CD86 and MHC-I for flow cytometric analysis of DC maturation.

### 2.13 T cells activation in vitro

CT26 cells were treated with PBS, TA/Fe, dBET57 or dBET@TF (at equivalent doses of 25 mg/L for dBET57 and 3.1 mg/L for Fe^3+^) for 24 h. Subsequently, the pretreated CT26 cells were co-cultured with murine CTLL2 lymphocytes for an additional 6 h. CTLL2 cells were then harvested and stained with antibodies against CD3, CD8 and IFNγ for flow cytometric analysis.

### 2.14 Biodistribution study of dEBT@TF in vivo

All experiments were conducted in strict accordance with the Institutional Animal Care and Use Committee (IACUC) of the Animal Experiment Center at Guangzhou Medical University (Approval No. GY2025-674) and with the Regulations for the Management of Laboratory Animals. To generate the xenograft model, 1 × 10^6^ CT26 cells were subcutaneously injected into the right dorsal flank of BALB/c mice (6 weeks old). 12 days after inoculation, when tumors reached about 100 mm^3^, animals were randomized into two groups. The first group was intravenously injected with Cy5.5-labeled dBET@TF (1.5 mg/kg Cy5.5), and the second group received free Cy5.5 at an equivalent dose and volume. Fluorescence imaging was performed using an in vivo imaging system at 0.5, 1.5, 3, 6, 9, 12, and 24 h post-injection. At 24 h after administration, animals were euthanized and their major organs excised for ex vivo fluorescence imaging, enabling quantitative assessment of biodistribution across the systemic circulation.

### 2.15 Antitumor effect of dBET@TF in vivo

CT26 tumor-bearing BALB/c mice were randomly allocated into five treatment cohorts. Mice received tail-vein injections of PBS, TA/Fe, dBET57 (4.1 mg/kg) or dBET@TF (containing 4.1 mg/kg dBET57 and 0.5 mg/kg Fe^3+^). Therapeutic dosing was administered once every two days for a total of three administrations. Throughout this period, body weight and tumor dimensions were measured every two days. At study termination, primary tumors and major organs were excised, fixed in 10 % neutral buffered formalin, and embedded in paraffin. Serial sections were stained for proliferation (Ki67), apoptosis (TUNEL) or routine histopathology (H&E). Major organs were similarly processed for H&E to assess off-target toxicity.

To establish an orthotopic CT26 tumor model, 5 × 10^5^ CT26 cells were mixed with 25 μL of Matrigel, and the mixture was injected into the cecal wall of each mouse. One week after tumor inoculation, mice received intravenous injections of PBS, TA/Fe, dBET57 (4.1 mg/kg), or dBET@TF (containing 4.1 mg/kg dBET57 and 0.5 mg/kg Fe^3+^) via the tail vein. Treatments were administered once every two days for a total of three doses. On day 14, mice were euthanized, and tumors were collected for volume measurement.

### 2.16 Analysis of immune cell infiltration

CT26 tumor-bearing mice were randomly assigned to five experimental groups and then intravenously injected with PBS, αPD-1 (2.5 mg/kg), TA/Fe combined with αPD-1 (2.5 mg/kg), dBET57 (4.1 mg/kg) combined with αPD-1 (2.5 mg/kg) or dBET@TF (containing 4.1 mg/kg dBET57 and 0.5 mg/kg Fe^3+^) combined with αPD-1 (2.5 mg/kg). Therapeutic dosing was administered once every two days for a total of three administrations. Two days following the last injection, mice were euthanized by cervical dislocation; tumor mass, inguinal lymph nodes, and spleens were excised, processed into single-cell suspensions, stained, and subjected to flow cytometric quantification of immune cell subsets.

### 2.17 Experimental lung metastasis suppression

A metastatic CT26 model was generated by tail-vein injection of 1 × 10^5^ CT26 cells into mice bearing orthotopic CT26 tumors. The following day, mice were intravenously injected with PBS, anti-PD-1 (2.5 mg/kg), TA/Fe in combination with αPD-1 (2.5 mg/kg), dBET57 alone (4.1 mg/kg) with αPD-1 (2.5 mg/kg) and dBET@TF (containing 4.1 mg/kg dBET57 and 0.51 mg/kg Fe^3+^) with αPD-1 (2.5 mg/kg). Therapeutic dosing was administered once every two days for a total of three administrations. Body weight and tumor dimensions were recorded bi-weekly during therapy. On day 13 post-injection, tumor and lung tissues were excised for downstream analyses.

### 2.18 Tumor rechallenge experiment

To evaluate the formation of long-term immunological memory, a tumor rechallenge experiment was performed. CT26 tumor-bearing mice were treated following the same regimen as described above. After the completion of treatment, the primary tumors were surgically removed. On day 21, 1 × 10^6^ CT26 cells were injected into the contralateral flank. Tumor growth was subsequently monitored every other day. On day 16 post-rechallenge, the mice were euthanized, and the tumors were excised and weighed.

### 2.19 Statistical analysis

All quantitative data are presented as mean ± standard deviation (SD) derived from a minimum of three independent replicates. Two-group comparisons were assessed with a two-tailed Student's t-test, whereas comparisons involving three or more groups employed a one-way analysis of variance (ANOVA). Statistical significance was assigned to P < 0.05.

## 3 Results and discussion

### 3.1 Preparation and characterization of dBET@TF

**Fig. 1:**
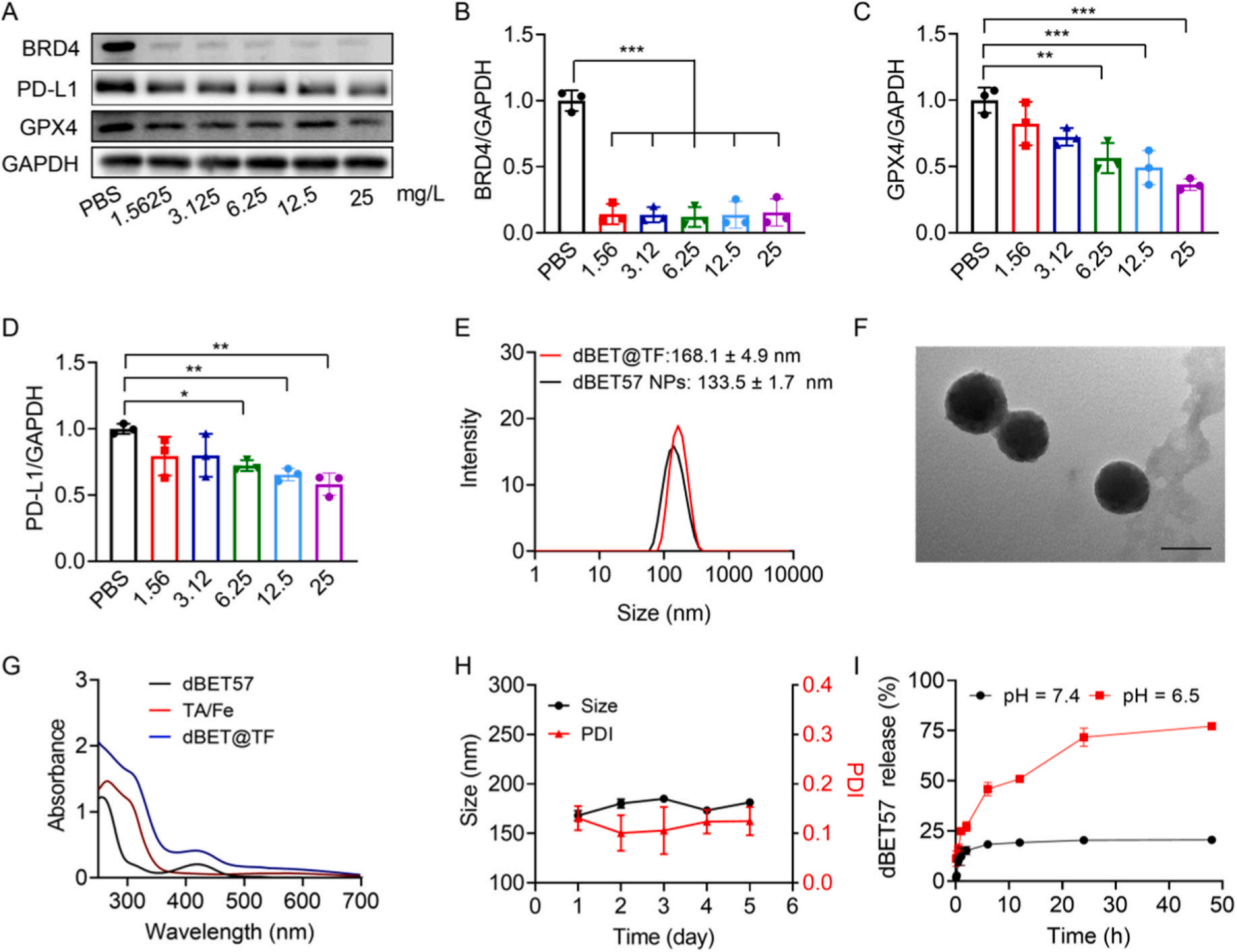
Preparation and characterization of dBET@TF. (A) Representative western blot membranes displaying the expression of BRD4, GPX4 and PD-L1. Quantitative densitometric analysis of (B) BRD4, (C) GPX4 and (D) PD-L1 proteins. (E) The particle sizes of dBET@TF and dBET57 nanoparticles. (F) TEM micrograph of dBET@TF, scale bar: 100 nm. (G) UV-Vis absorption spectra of dBET57, TA/Fe, and dBET@TF. (H) Particle size and PDI changes of dBET@TF over 5 days. (I) In vitro drug release profiles of dBET@TF under physiological (pH 7.4) and acid (pH 6.5) conditions, quantified as cumulative dBET57 release. Statistical significance was indicated as *P < 0.05, **P < 0.01 and ***P < 0.001.

We first assessed the capacity of dBET57 to induce BRD4 degradation, which in turn led to transcriptional suppression of GPX4 and PD-L1. As presented in Fig. 1A, dBET57 potently mediated BRD4 degradation, achieving over 85 % depletion of BRD4 protein even at the lowest tested concentration of 1.56 mg/L (Fig. 1B). Concomitant with BRD4 downregulation, the expression of PD-L1 and GPX4 was markedly suppressed (Fig. 1C and D). At the maximum dBET57 concentration of 25 mg/L, GPX4 and PD-L1 levels decreased to 36.3 % and 58.2 % of their initial amounts, respectively. Given the critical roles of GPX4 in preventing ferroptosis initiation by detoxifying lipid peroxides and of PD-L1 in mediating immune escape, this dual downregulation highlighted significant therapeutic potential of dBET57 for potentiating colorectal cancer immunotherapy.

Capitalizing on the therapeutic advantages of dBET57, we proceeded to engineer self-assembled nanomedicines incorporating Fe^3+^, tannic acid (TA) and dBET57. As displayed in Fig. S1 and 1E, dBET57 readily self-assembled into homogeneous spherical nanoparticles with a size of 133.5 ± 1.7 nm in aqueous media. The subsequent addition of Fe^3+^ and TA led to a size increase to 168.1 ± 4.9 nm, suggesting the formation of an TA/Fe network encapsulating the dBET57 nanoparticles (Fig. 1F). To definitively confirm the encapsulation, the entrapped dBET57 was extracted using DMF. The solvent-caused etching of dBET@TF yielded hollow TA/Fe network with a shell thickness of approximately 13 nm (Fig. S2), validating the encapsulation strategy. High-performance liquid chromatography (HPLC) analysis confirmed an efficient entrapment efficiency of dBET57 within dBET@TF, reaching 47.3 %, indicative of effective drug loading (Fig. S3). Furthermore, UV-Vis spectroscopy revealed that dBET@TF exhibited characteristic absorption peaks of both dBET and the TA/Fe complex (Fig. 1G). Collectively, these findings demonstrated that dBET57 nanoparticles were effectively encapsulated within the TA/Fe network, a structural integrity that was expected to impart enhanced colloidal stability and stimuli-responsive drug release characteristics to dBET@TF. Subsequently, the colloidal stability of dBET@TF was evaluated, showing sustained particle size and polydispersity index (PDI) values over a 5-day period in aqueous buffer, underscoring its potential for biomedical applications (Fig. 1H). The stimuli-responsive drug release profile of dBET@TF was further investigated. As illustrated in Fig. 1I, dBET57 release was minimal (20.6 %) in a neutral buffer. By contrast, under acidic conditions (pH 6.5), the release rate drastically increased to 77.2 %. This pronounced pH-responsive drug release highlighted the potential for targeted tumor delivery and a corresponding reduction in off-target effects. Additionally, we evaluated the “hook effect” of dBET@TF, a phenomenon in which excess binary PROTAC-target or PROTAC-E3 ligase complexes compete with productive ternary complex formation, thereby compromising degradation efficiency at high concentrations. CT26 cells were treated with various concentrations of dBET@TF. As shown in Fig. S4, even at the maximum tested concentration, dBET@TF did not exhibit the classic hook effect, indicating that the acid-responsive drug release property of dBET@TF did not compromise its therapeutic index.

### 3.2 Cytotoxicity of dBET@TF in vitro

**Fig. 2:**
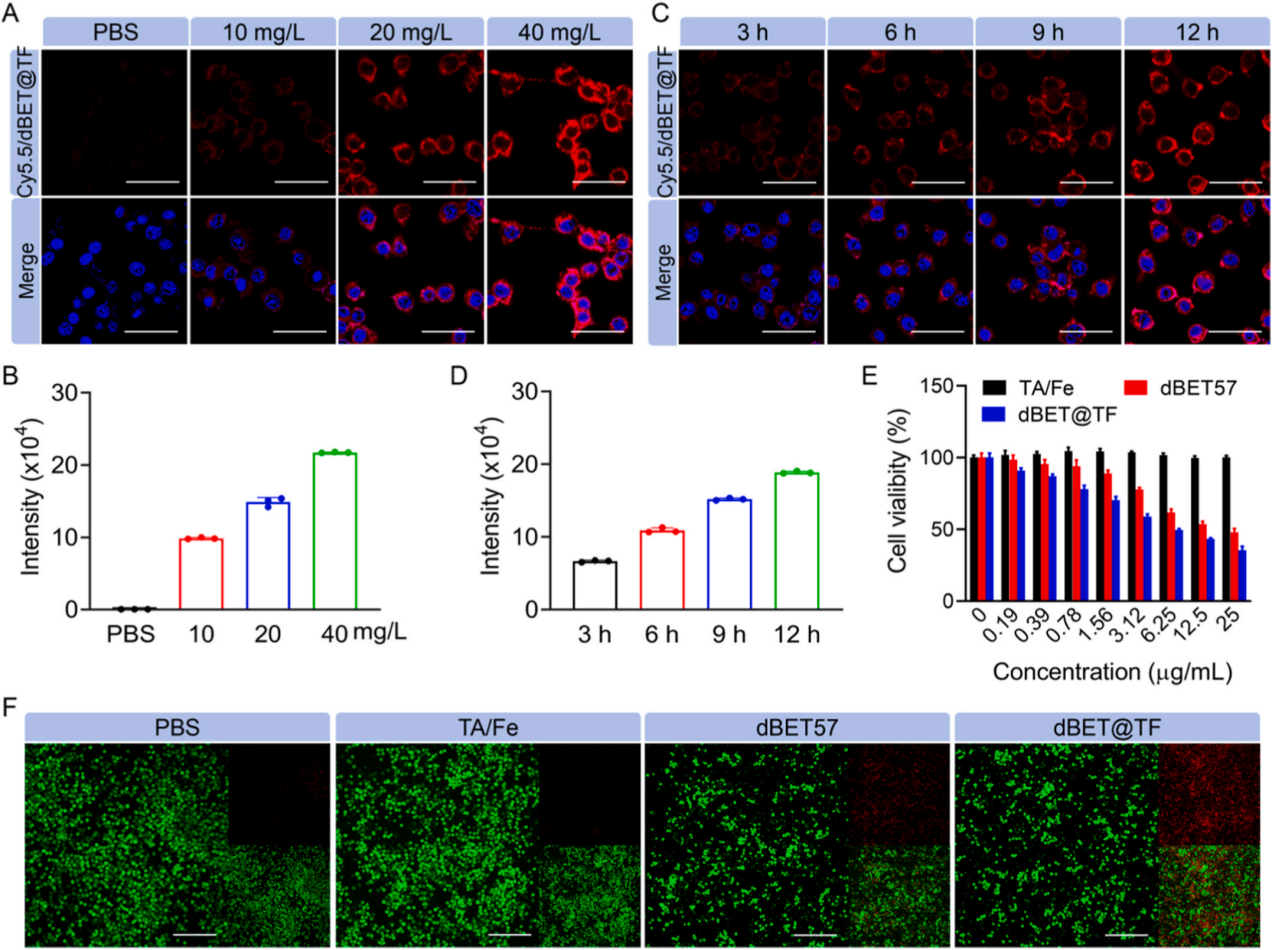
Antitumor effect of dBET@TF in vitro. (A) Representative micrographs and (B) fluorescence quantitative analysis of CT26 cells after 24 h incubation with the indicated concentrations of dBET@TF. Scale bar: 50 μm. (C) Representative micrographs and (D) fluorescence quantitative analysis of CT26 cells incubated with dBET@TF for various times. Scale bar: 50 μm. (E) MTT viability assay of CT26 cells incubated with gradient concentrations of TA/Fe, dBET57 and dBET@TF. (F) Live/dead cells staining of CT26 cells after 24 h incubation with PBS, TA/Fe, dBET57 and dBET@TF for 24 h. Scale bar: 200 μm.

Before evaluating the cytotoxic efficacy of dBET@TF, we characterized its cellular internalization behavior using CLSM. For these studies, dBET@TF was labeled with Cy5.5 to generate Cy5.5/dBET@TF, which was then co-incubated with murine colorectal cancer (CT26) cells. As evidenced by the fluorescence microscopy images and quantitative analysis of fluorescence intensity (Fig. 2A–D and S5), Cy5.5/dBET@TF demonstrated a notable, concentration- and time-dependent cellular uptake. The observed gradual increase in fluorescence intensity with escalating nanomedicine concentration and prolonged incubation durations strongly suggested efficient internalization of the nanomedicine by CT26 cells. This efficient cellular uptake formed a crucial foundation for its potential therapeutic efficacy. To elucidate the cellular uptake pathway of dBET@TF, CT26 cells were pretreated with specific pharmacological inhibitors targeting distinct endocytic routes. As shown in Fig. S6, treatment with Pitstop 2, a clathrin-mediated endocytosis inhibitor, markedly reduced the internalization efficiency of dBET@TF. Methyl-β-cyclodextrin (M-β-CD), which disrupts caveolae-mediated endocytosis, also inhibited dBET@TF uptake, albeit to a lesser extent. In contrast, 5-(N-ethyl-N-isopropyl) amiloride (EIPA), an inhibitor of macropinocytosis, had only a minimal effect on dBET@TF internalization. Collectively, these results indicated that clathrin- and caveolae-mediated endocytosis serve as the primary uptake mechanisms for dBET@TF.

Subsequently, the anti-proliferative effects of dBET@TF were evaluated using MTT assays and live/dead cell staining. The TA/Fe mixture alone exhibited minimal intrinsic cytotoxicity against CT26 cells, with cell viability remaining largely unchanged across the tested concentrations (Fig. 2E). In contrast, free dBET57 induced dose-dependent cytotoxicity, an effect linked specifically to BRD4 degradation. Notably, dBET@TF significantly outperformed both free dBET57 and the TA/Fe carrier. At the highest concentration tested, dBET@TF treatment resulted in substantial growth inhibition, reducing cell viability to 35.4 %. This enhanced anti-proliferative efficacy was further corroborated by live/dead cell staining (Fig. 2F). Cells treated with dBET@TF exhibited the most intense red fluorescence, indicating extensive cell death. Quantitative analysis of PI fluorescence intensity further supported these findings, revealing a 4.5-fold increase in the dBET@TF group relative to the PBS group (Fig. S7). The observed therapeutic enhancement likely arose from the synergistic effect of iron accumulation combined with dBET57-mediated BRD4 degradation.

### 3.3 Ferroptosis amplification by dBET@TF

**Fig. 3:**
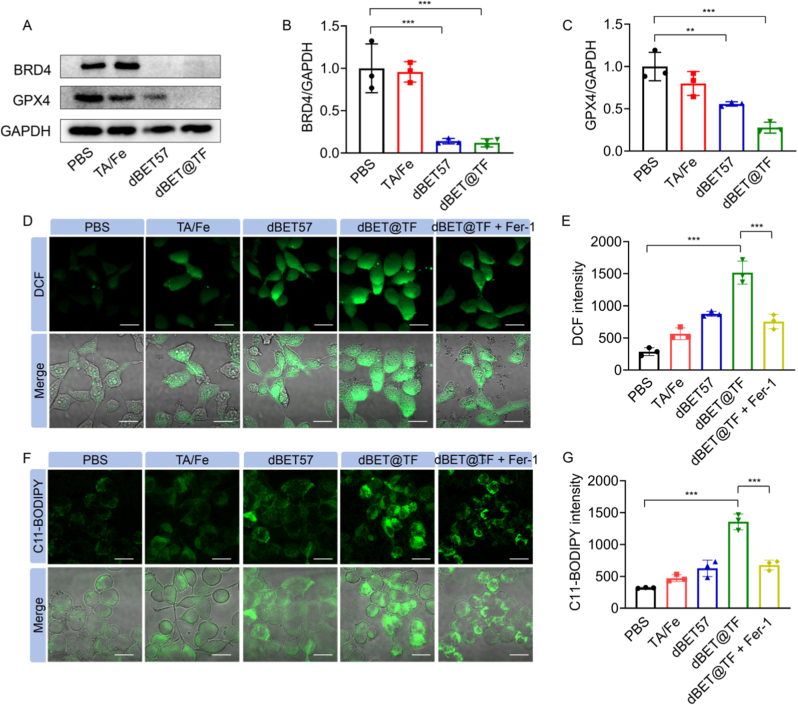
Ferroptosis amplification by dBET@TF. (A) Representative Western blot profiles of BRD4 and GPX4. Quantitative densitometric analysis of (B) BRD4 and (C) GPX4 proteins. (D) Fluorescent imaging of intracellular ROS in CT26 cells treated with PBS, TA/Fe, dBET57, dBET@TF and dBET@TF + Fer-1 for 24 h. Scale bars: 20 μm. (E) Flow cytometry analysis of CT26 cells after treatment with PBS, TA/Fe, dBET57, dBET@TF and dBET@TF + Fer-1 for 24 h. (F) Lipid peroxide production in CT26 cells after treatment with PBS, TA/Fe, dBET57 and dBET@TF for 24 h. (G) Quantitative fluorescence analysis of lipid peroxide levels. 20 μm. Statistical significance was indicated as **P < 0.01 and ***P < 0.001.

Ferroptosis is a form of regulated cell death driven by iron-dependent accumulation of lipid peroxides [40,41]. To determine whether the potent antitumor effects of dBET@TF are mediated through ferroptosis, we systematically evaluated key molecular markers associated with this pathway. Results showed that a modest reduction in GPX4 expression was observed following TA/Fe treatment, whereas dBET57-mediated BRD4 degradation led to substantial downregulation (Fig. 3A). In contrast, dBET@TF treatment resulted in near-complete GPX4 depletion, with a reduction exceeding 72 % (Fig. 3B and C), confirming potent ferroptosis induction. To rule out the involvement of parallel ferroptosis defense pathways, we examined the expression of FSP1, a key ferroptosis suppressor that functions independently of the GPX4 axis. As shown in Fig. S8, neither dBET57 nor dBET@TF exerted any detectable effect on FSP1 protein expression, indicating that the ferroptosis-sensitizing effect of BRD4 degradation was specifically mediated through GPX4 downregulation. We next evaluated intracellular ROS levels. Confocal microscopy revealed weak green fluorescence in cells treated with TA/Fe or dBET57 alone, consistent with minimal ROS generation (Fig. 3D). Conversely, dBET@TF treatment led to a pronounced increase in green fluorescence. Fluorescence quantitative analysis demonstrated that the mean fluorescence intensity in the dBET@TF group was 5.2-fold higher than that in the PBS group (Fig. 3E), indicating substantial ROS elevation. To directly assess lipid peroxidation, we employed the C11-BODIPY probe. Confocal imaging revealed markedly brighter green fluorescence in the dBET@TF-treated group (Fig. 3F), indicative of enhanced lipid peroxidation. This finding was quantitatively corroborated, with the dBET@TF group exhibiting the highest fluorescence intensity (Fig. 3G). Notably, dBET@TF-induced ROS production and lipid peroxidation were markedly attenuated upon co-treatment with ferrostatin-1, a well-established ferroptosis inhibitor, confirming that these effects originated specifically from dBET@TF-mediated ferroptosis. Collectively, these results demonstrated that dBET@TF induced substantial LPO and ROS accumulation alongside profound GPX4 suppression, establishing ferroptosis as a key mechanism underlying its potent antitumor activity.

### 3.4 dBET@TF-mediated ICD effect and PD-L1 downregulation

**Fig. 4:**
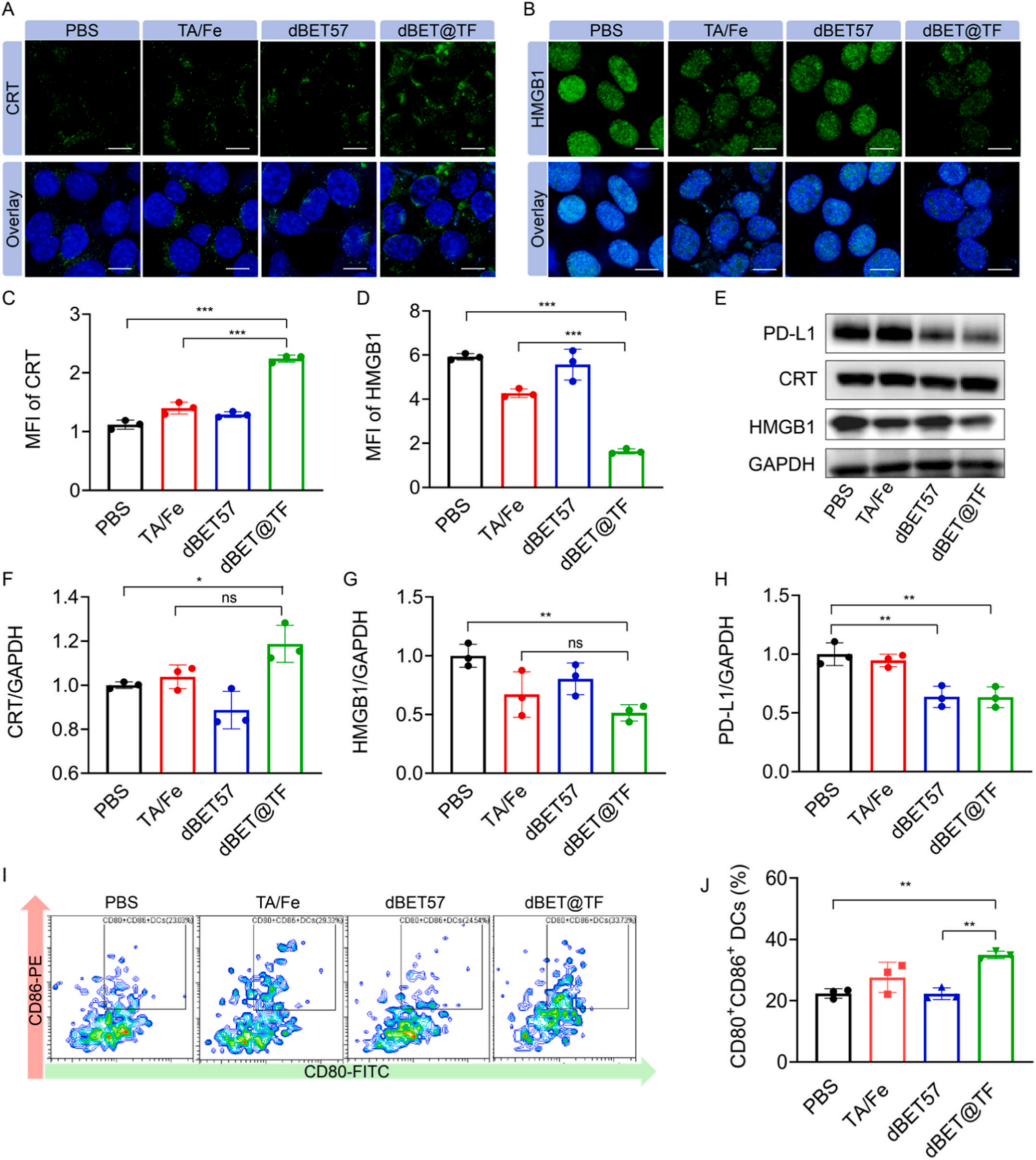
dBET@TF-mediated ICD effect and PD-L1 downregulation. (A) CRT exposure of CT26 cells after exposure to PBS, TA/Fe, dBET57 and dBET@TF for 24 h. Scale bar: 20 μm. (B) HMGB1 release of CT26 cells after treatment with PBS, TA/Fe, dBET57 and dBET@TF for 24 h. Scale bar: 20 μm. (C) Fluorescence-based quantification of CRT protein. (D) Fluorescence-based quantification of HMGB1 protein. (E) Representative western blot bands. Densitometric quantification of (F) CRT, (G) HMGB1, and (H) PD-L1 proteins. (I) Representative flow cytometry plots illustrating CD80^+^CD86^+^ DCs. (J) Quantification of the percentage of CD80^+^CD86^+^ DCs following treatment with the indicated agents. Statistical significance was indicated as *P < 0.05, **P < 0.01 and ***P < 0.001.

To investigate whether dBET@TF-induced ferroptosis triggers an ICD response, immunofluorescence staining was carried out to assess the redistribution of calreticulin (CRT) and high-mobility group box 1 (HMGB1). Confocal imaging revealed a marked increase in green fluorescence intensity at the plasma membrane in dBET@TF-treated cells, indicating surface exposure of CRT (Fig. 4A). Concurrently, HMGB1 exhibited complete nuclear depletion and extracellular release in dBET@TF-treated cells, whereas control groups retained strong nuclear localization (Fig. 4B). Quantitative analysis showed a 2.0-fold increase in CRT surface exposure and a 0.7-fold reduction in intracellular HMGB1 compared with the PBS group (Fig. 4C and D). Moreover, extracellular ATP levels were also measured under various treatment conditions. Compared with the PBS group, dBET@TF treatment led to a 2-fold increase in ATP secretion into the culture medium (Fig. S9). The coordinated release of HMGB1 and ATP, together with CRT exposure, identified dBET@TF as a potent ICD inducer that acted through ferroptotic cell death. To further validate these findings, we conducted parallel western blot analyses (Fig. 4E–G). Consistent with the imaging data, dBET@TF treatment led to a 1.2-fold increase in total CRT expression and a 0.5-fold reduction in HMGB1 protein levels, confirming the loss of intracellular DAMPs and their pro-immunogenic mobilization. We next investigated whether the DAMPs released from dBET@TF-treated tumor cells could promote the maturation of dendritic cells (DCs). CT26 cells pretreated with dBET@TF were co-cultured with bone marrow-derived dendritic cells (BMDCs). As shown in Fig. 4I and J, the percentage of CD80^+^CD86^+^ DCs in the PBS group was 22.3 %, whereas this value was significantly elevated to 34.9 % in the dBET@TF-treated group. Moreover, MHC-I expression was markedly upregulated when BMDCs were co-cultured with dBET@TF-treated CT26 cells (Fig. S10). These results demonstrated that dBET@TF-induced DAMPs release effectively promoted the maturation and antigen-presenting capacity of BMDCs, thereby facilitating the activation of antitumor immunity.

Importantly, dBET@TF also reduced PD-L1 expression by more than 35 % (Fig. 4H), suggesting its capacity not only to induce ICD but also to counteract tumor-mediated immune evasion. To investigate whether downregulation of PD-L1 on CT26 cells could reverse T lymphocyte exhaustion, CT26 cells pretreated with dBET@TF were co-cultured with murine lymphocyte CTLL2 cells, with untreated CTLL2 cells serving as the control. As shown in Fig. S11, in the PBS group, the percentage of CD3^+^TIM3^+^ exhausted T cells increased to 8.2 %, indicative of progressive T cell dysfunction under PD-L1-competent conditions. In sharp contrast, this exhausted population was significantly diminished in the dBET@TF group, with the percentage of CD3^+^TIM3^+^ T cells restored to levels comparable with the untreated control, demonstrating that dBET@TF-mediated PD-L1 degradation effectively attenuated T cell exhaustion. Concomitantly, the percentage of CD8^+^ IFNγ^+^ T cells was markedly elevated (Fig. S12), indicating that dBET@TF-mediated ferroptotic cell death, coupled with PD-L1 degradation, effectively alleviated T cell exhaustion and promotes the activation of CD8^+^ T cells. Together, these findings established dBET@TF as a dual-function therapeutic agent that simultaneously activated antitumor immunity and inhibited key immune checkpoints, positioning it as a promising platform for synergistic combination with checkpoint inhibitors.

### 3.5 In vivo antitumor effect of dBET@TF

**Fig. 5:**
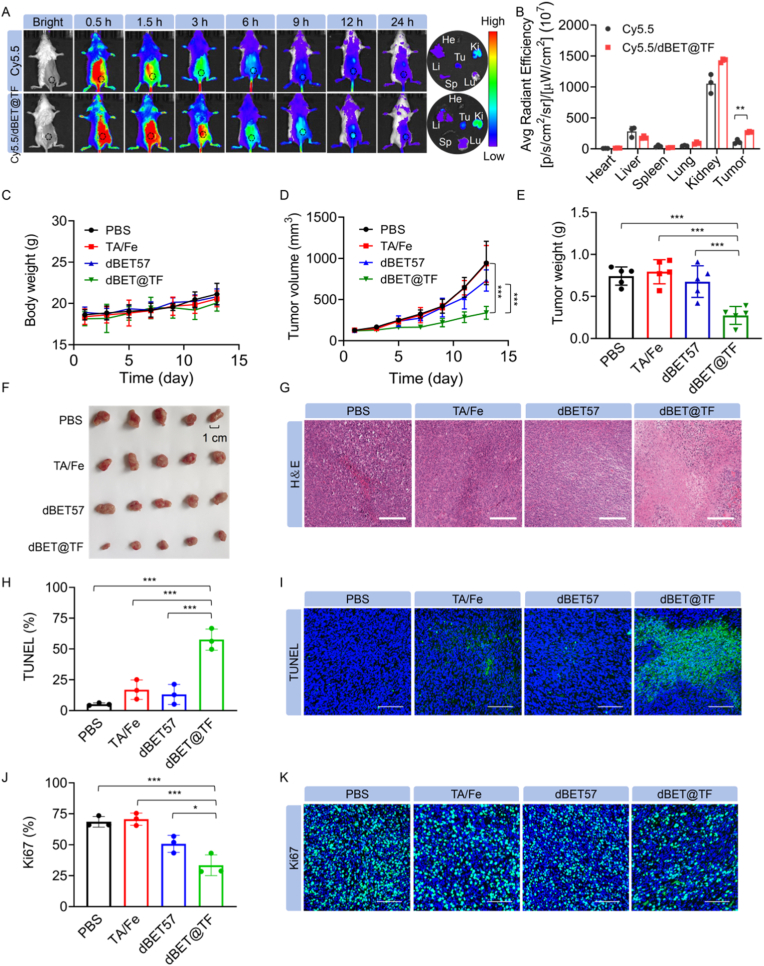
*In vivo* antitumor effect of dBET@TF. (A) Biodistribution of dBET@TF in CT26 tumor-bearing mice. (B) Quantification of the fluorescence signal in isolated organs. (C) Body weights, (D) tumor volumes, (E) tumor weights and (F) representative tumor images of CT26 tumor-bearing mice after intravenous injection of PBS, TA/Fe, dBET57, and dBET@TF. (G) H&E-stained tumor sections. Scale bar: 100 μm. (H) TUNEL-positive rate and (I) representative images of TUNEL staining in tumor sections. Scale bar: 100 μm. (J) Ki67-positive rate and (K) representative images of Ki67 staining in tumor sections. Scale bar: 100 μm. Statistical significance was indicated as *P < 0.05 and ***P < 0.001.

Nanomedicines exploit the enhanced permeability and retention (EPR) effect for passive tumor targeting, which boosts therapeutic payload delivery and limits off-target toxicity [[42], [43], [44]]. To assess the in vivo tumor-homing efficacy of dBET@TF, we administered Cy5.5-labeled dBET@TF intravenously to CT26-bearing mice and monitored its biodistribution by fluorescence imaging. Systemically injected Cy5.5/dBET@TF showed widespread distribution and sustained blood circulation (Fig. 5A), with fluorescence progressively enriching at the tumor site over time. Examination of excised tumor tissues confirmed a pronounced enrichment of Cy5.5 signal in the Cy5.5-labeled dBET@TF group, whereas the free Cy5.5 group displayed only marginal fluorescence. Quantification revealed that the fluorescence intensity in tumor tissue from mice treated with Cy5.5/dBET@TF was 2.4-fold higher than that in the free Cy5.5 group (Fig. 5B), confirming effective passive targeting and selective tumor enrichment.

To further assess the therapeutic potential of dBET@TF, we systematically evaluated its antitumor efficacy in a murine CT26 colorectal cancer model. Mice were intravenously administered PBS, TA/Fe, dBET57, or dBET@TF, and body weight was recorded throughout the course of treatment as an indicator of systemic tolerability. All groups maintained a steady gain in body weight, indicating that dBET@TF exhibited good biocompatibility and causes negligible systemic toxicity (Fig. 5C). Concerning antitumor efficacy, the PBS-treated mice demonstrated rapid tumor expansion, culminating in an average final volume of 943.9 mm^3^. The TA/Fe group did not differ significantly from the control, indicating limited therapeutic benefit from iron delivery alone. Treatment with dBET57 monotherapy resulted in modest tumor growth inhibition. In contrast, dBET@TF induced marked suppression of tumor growth, reducing the final tumor volume to 338.3 mm^3^, representing a 64.2 % reduction relative to PBS group (Fig. 5D). Tumor weight measurements and imaging corroborated these findings; tumors treated with dBET@TF weighed only 274 mg, significantly less than those in all other groups (Fig. 5E and F). The pronounced antitumor efficacy of dBET@TF highlighted the synergistic therapeutic effect achieved through combined GPX4 suppression and iron accumulation. Mechanistic investigations revealed extensive necrotic zones in H&E-stained sections (Fig. 5G), indicative of irreversible cell death driven by synergistic ferroptosis and BRD4 degradation. TUNEL assay demonstrated a striking increase in apoptotic cells, with 57.6 % of nuclei exhibiting DNA fragmentation (Fig. 5H and I). Immunofluorescence staining for Ki67 revealed a dramatic reduction in proliferative activity, with only 33.4 % of cells positive for Ki67 (Fig. 5J and K), confirming potent anti-proliferative effects. Importantly, comprehensive histopathological and biochemical assessments revealed no treatment-related abnormalities in major organs following dBET@TF administration. H&E staining of major organs showed no discernible inflammatory infiltration, necrosis, or structural distortion (Fig. S13). Furthermore, serum levels of liver function markers (ALT and AST), as well as renal function indicators (UA and BUN), remained within normal physiological ranges (Fig. S14). These findings collectively indicated that dBET@TF exhibited a favorable biosafety profile with minimal systemic toxicity.

To evaluate the antitumor efficacy of dBET@TF in a more physiologically representative model, an orthotopic tumor model was established. As shown in Fig. S15, tumors in the PBS-treated control group exhibited aggressive growth, reaching a mean volume of 1131 mm^3^ by the study endpoint. In contrast, treatment with the non-targeted components dBET57 or TA/Fe alone failed to appreciably retard tumor proliferation. Notably, dBET@TF exerted a robust antitumor effect, significantly suppressing solid tumor growth throughout the treatment period. This pronounced therapeutic efficacy reaffirmed the synergistic benefit achieved through the combination of BRD4-targeted GPX4 suppression and iron-mediated ferroptosis amplification. Collectively, these findings established dBET@TF as a highly efficient, biocompatible, and synergistically potent nanotherapeutic platform capable of co-delivering BRD4 degraders and iron for the targeted treatment of colorectal cancer.

### 3.6 Potentiating αPD-1 therapy by dBET@TF

**Fig. 6:**
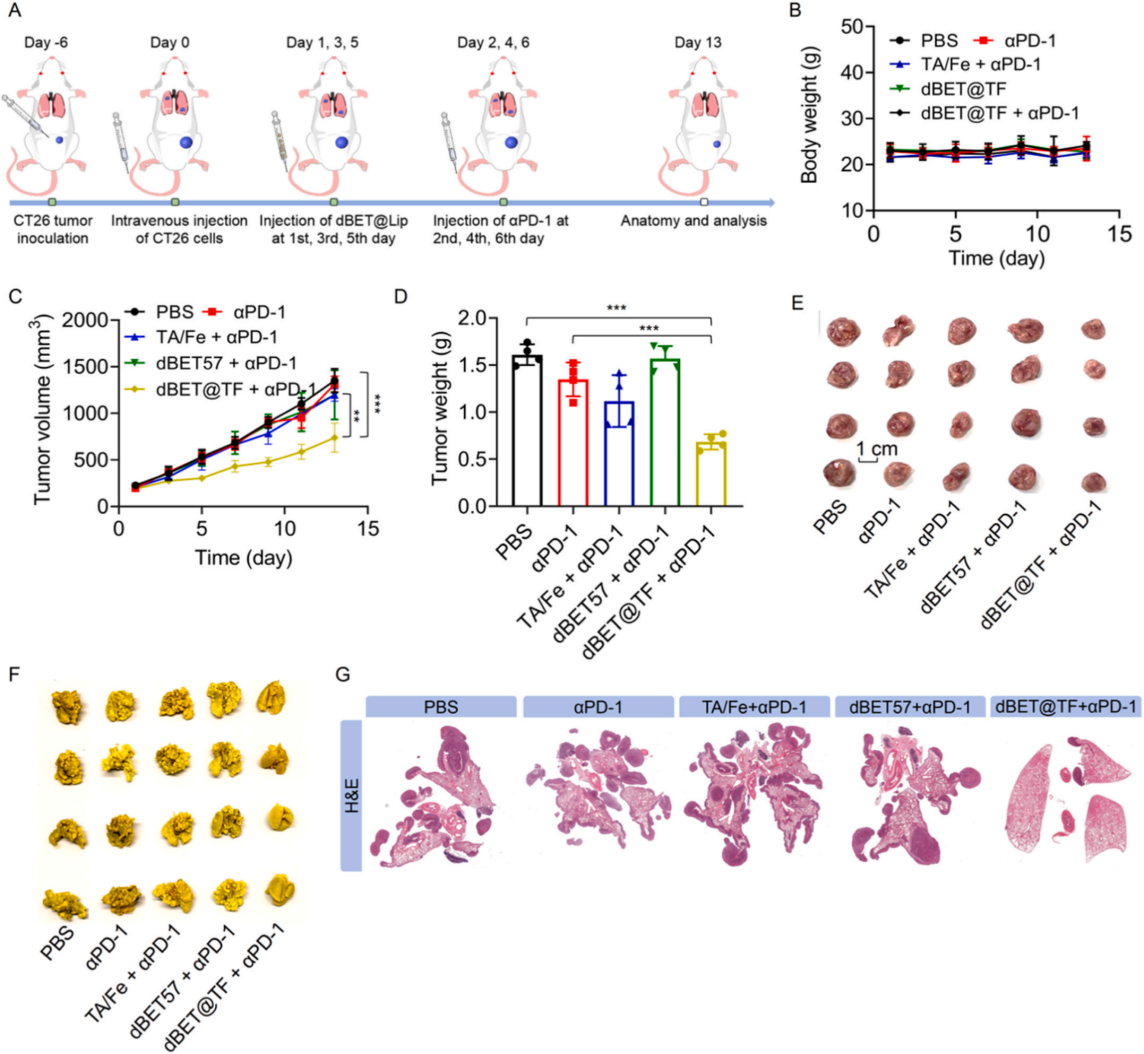
Potentiating αPD-1 therapy by dBET@TF. (A) Schematic diagram of treatment regimens. (B) Body weights, (C) tumor volumes, (D) tumor weights and (E) representative tumor images of CT26 tumor-bearing mice after intravenous injection of PBS, αPD-1, TA/Fe + αPD-1, dBET57 + αPD-1, and dBET@TF + αPD-1. (F) Gross photographs of harvested lungs and (G) H&E stained lung sections illustrating metastatic burden. Statistical significance was indicated as **P < 0.01 and ***P < 0.001.

The efficacy of dBET@TF in suppressing experimental lung metastasis was subsequently examined using a tail-vein injection lung colonization model (Fig. 6A). Over the full course of treatment, none of the groups exhibited appreciable body weight loss (Fig. 6B), confirming the favorable biosafety and tolerability of all formulations. Notably, dBET@TF exhibited synergistic antitumor activity when combined with αPD-1. As shown in Fig. 6C and D, combination therapy reduced primary tumor volume to 54.6 % and tumor weight to 42.5 % of the values observed in the PBS group, significantly outperforming other therapies. Tumor imaging further confirmed the most pronounced antitumor response in the dBET@TF + αPD-1 group, with visibly smaller tumors compared with all other treatments (Fig. 6E). Crucially, only the dBET@TF + αPD-1 regimen effectively suppressed metastatic colonization in the lung. H&E analysis of lung sections revealed a pronounced infiltrate of tumor cells in the PBS-treated group. In contrast, the combination-treated group showed markedly fewer metastatic nodules, indicating effective inhibition of systemic spread (Fig. 6F and G). Of note, neither TA/Fe + αPD-1 nor dBET57 + αPD-1 significantly reduced metastasis, underscoring the necessity of both BRD4 degradation and iron-mediated ferroptosis for achieving immunogenic tumor killing. Taken together, these results underscored dBET@TF as a powerful immunostimulatory nanotherapeutic that, in synergy with αPD-1 blockade, concurrently restrained primary tumor growth and inhibited experimental lung metastasis.

To investigate whether dBET@TF + αPD-1 combination therapy could induce the formation of long-term immunological memory, we performed a tumor rechallenge experiment in surviving mice. As illustrated in Fig. S16A, mice that had received the indicated treatments were rechallenged on day 22 via contralateral subcutaneous implantation of CT26 cells. In striking contrast to the aggressive tumor growth observed in the PBS-treated group, tumor progression was profoundly suppressed in the dBET@TF + αPD-1 group, with the mean tumor volume reaching only 103 mm^3^. Consistently, analysis of tumor weight and representative images of excised tumors further confirmed the durable therapeutic efficacy of this combination regimen, with the mean tumor weight in the dBET@TF + αPD-1 group being merely 0.09-fold that of the PBS group (Fig. S16B and S16C). These data provided strong evidence for the establishment of long-term immunological memory following combinatorial therapy. To further validate the generation of a memory T cell response, spleens were harvested from rechallenged mice for immunophenotypic analysis (Fig. S17). Flow cytometric quantification revealed a significantly elevated proportion of central memory T cells within the CD8^+^ T cell population in the dBET@TF + αPD-1 group compared with the PBS group, indicating successful induction of durable T cell memory. Collectively, these results substantiated that the synergistic therapeutic modality combining enhanced ferroptosis induction with immune checkpoint blockade not only eradicated established tumors but also established protective immunological memory against tumor recurrence.

### 3.7 Antitumor immune activation of dBET@TF + αPD-1

**Fig. 7:**
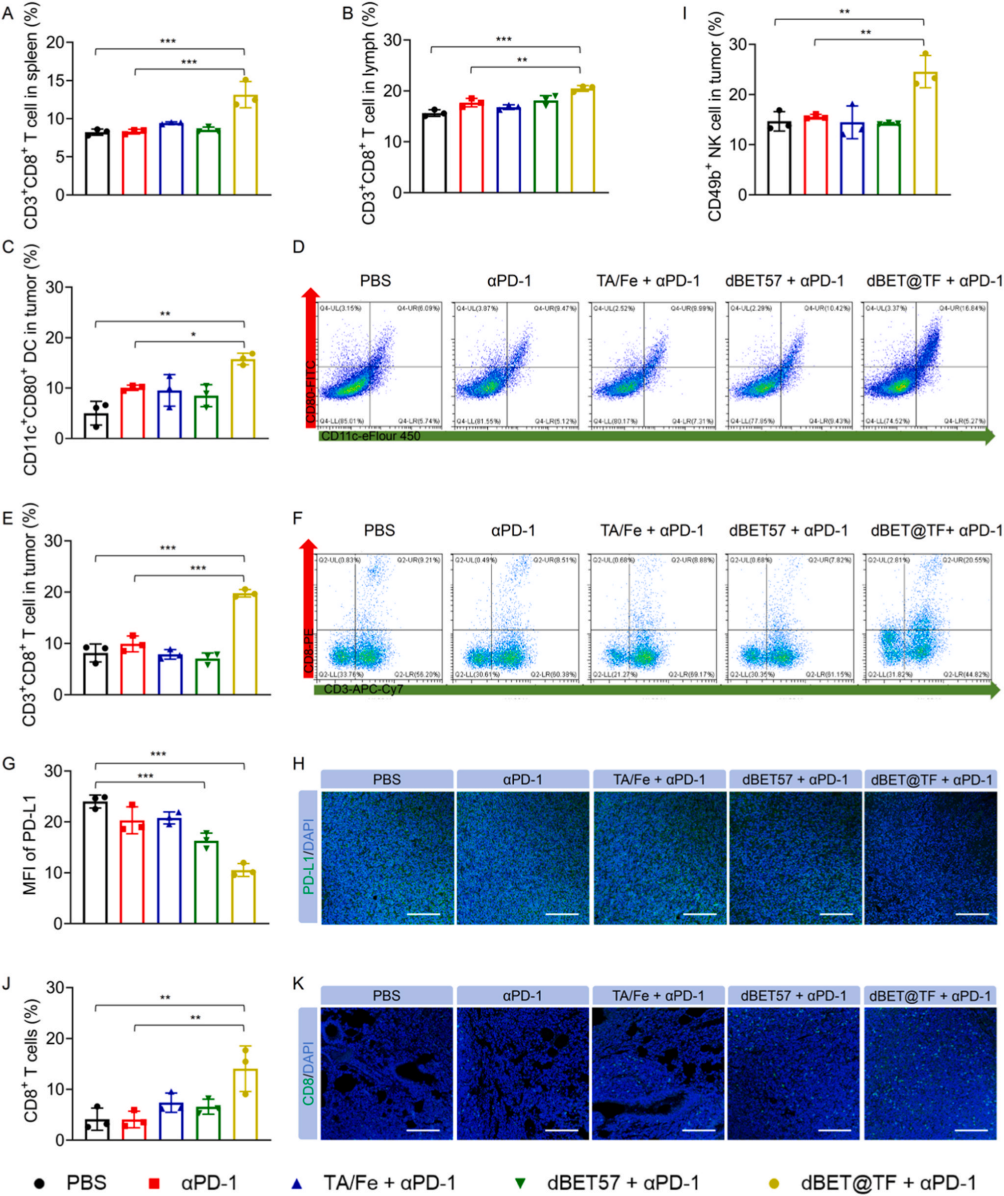
Antitumor immune activation by dBET@TF + αPD-1. (A) Proportion of CD3^+^CD8^+^ T cells within draining lymph nodes and (B) spleen after treatment with PBS, αPD-1, TA/Fe + αPD-1, dBET57 + αPD-1, and dBET@TF + αPD-1. (C) Proportion of CD11c^+^CD80^+^ DCs residing in tumor tissue following the same treatment regimens. (D) Representative flow cytometry plots illustrating CD11c^+^CD80^+^ DCs in tumor tissues. (E) Relative abundance of tumor resident CD3^+^CD8^+^ T cells. (F) Representative flow cytometry plots illustrating CD3^+^CD8^+^ T cells in tumor tissue. (G) Fluorescence-based quantification of PD-L1 expression. (H) Immunofluorescence micrographs depicting PD-L1 localization in CT26 tumor sections obtained after treatment with PBS, αPD-1, TA/Fe + αPD-1, dBET57 + αPD-1, and dBET@TF + αPD-1. Scale bar: 100 μm. (I) Proportion of CD49b^+^ NK cells within tumor tissues. (J) Proportion of CD8^+^ T cells in lung tissues. (K) CD8 immunofluorescence of lung tissues. Scale bar: 100 μm. Statistical significance was indicated as *P < 0.05, **P < 0.01 and ***P < 0.001.

To dissect the immunologic basis of the enhanced antimetastatic efficacy observed with the dBET@TF + αPD-1 regimen, comprehensive flow cytometry and immunofluorescence analyses were conducted. The combination therapy markedly amplified the frequencies of CD3^+^ CD8^+^ cytotoxic T lymphocytes and CD3^+^ CD4^+^ helper T cells within both the spleen and lymph nodes, underscoring a robust systemic T-cell activation that likely underpinned the enhanced therapeutic effect (Fig. 7A and B and S18). Within the tumor microenvironment, while combination therapies involving either TA/Fe + αPD-1 or dBET57 + αPD-1 failed to elicit significant maturation of tumor-resident dendritic cells (DCs), the dBET@TF + αPD-1 regimen markedly enhanced DC maturation. This observation suggested that dBET@TF-mediated ICD promoted antigen presentation and subsequent T cell priming (Fig. 7C and D). Furthermore, the combination therapy led to a marked increase in CD3^+^CD8^+^ T cell infiltration, rising from 8.1 % in PBS group to 19.7 % in the dBET@TF + αPD-1 group (Fig. 7E and F). CD8 immunofluorescence confirmed a 2-fold increase in CD8^+^ cells, indicating that dBET@TF promoted CTL recruitment and activation, thereby augmenting ICB efficacy (Fig. S19). To further assess whether CD8^+^ T cells in the tumor microenvironment possess sustained cytotoxic functionality, we performed intracellular staining for IFNγ and TNFα (Fig. S20). The results revealed that the percentages of CD8^+^ IFNγ^+^ and CD8^+^ TNFα^+^ T cells were significantly elevated following treatment with dBET@TF + αPD-1, indicating a robust and polyfunctional cytotoxic T cell response. Furthermore, dBET@TF demonstrably reduced PD-L1 expression on tumor cells (Fig. 7G and H), a critical step in mitigating immune evasion and overcoming adaptive resistance. In addition to T cell responses, the involvement of innate immune cells was investigated. While TA/Fe + αPD-1 and dBET57 + αPD-1 yielded only modest NK cell increases, dBET@TF + αPD-1 induced a 1.6-fold infiltration of NK cells, reaching 24.5 %, validating its capacity to broaden immune engagement (Fig. 7I). Systemic immune activation extended to distal metastatic sites, with robust CD8^+^ T cell infiltration observed in the lungs of dBET@TF + αPD-1 treated mice (Fig. 7J and K). These findings collectively underscored that dBET@TF synergized with αPD-1 blockade by orchestrating coordinated adaptive and innate immunity initiated by PD-L1 downregulation and ferroptosis-mediated ICD response. Its distinct ability to amplify ICB efficacy positioned dBET@TF as a potent immunostimulant for enhancing therapeutic outcomes in colorectal cancer.

## 4 Conclusion

In summary, we engineered an acid-responsive PROTAC-incorporated immunostimulant, termed dBET@TF, which amplified ferroptotic cell death and potentiated ICB therapy through the concurrent downregulation of PD-L1 and GPX4. Specifically, dBET@TF was formulated by self-assembly of dBET57, tannic acid, and Fe^3+^, a strategy that markedly improved drug solubility and the intracellular delivery of therapeutic agents. Mechanistically, dBET@TF drived ferroptotic cell death through iron overload and GPX4 degradation, thereby eliciting a potent ICD response. Crucially, concurrent PD-L1 downregulation reversed T-cell exhaustion and promoted robust infiltration of NK cells and CD8^+^ T cells. Consequently, dBET@TF synergized with αPD-1 to significantly suppress primary tumor growth and inhibit experimental lung metastasis, while also establishing protective immunological memory against tumor rechallenge. Overall, this work established PROTAC-based combinatorial strategies as a promising immunostimulatory platform capable of mounting antitumor immunity and enhancing the response to ICB in colorectal cancer.

## CRediT authorship contribution statement

**Xueping Luo:** Investigation, Methodology, Writing – original draft. **Yixin Liu:** Investigation, Methodology. **Guangmiao Chen:** Investigation, Methodology. **Xinrui Ma:** Investigation. **Pingping Xu:** Investigation. **Yutong Li:** Investigation. **Longyu Zheng:** Investigation. **Rongrong Zheng:** Investigation. **Youqin Xu:** Investigation. **Shiying Li:** Supervision. **Linping Zhao:** Conceptualization, Funding acquisition, Project administration, Supervision, Writing – review & editing.

## Declaration of competing interest

The authors declare that they have no known competing financial interests or personal relationships that could have appeared to influence the work reported in this paper.

## Data Availability

Data will be made available on request.
