# Supplementary material for: Self-assembled nano-PROTACs potentiate colorectal cancer immunotherapy via downregulating PD-L1 and GPX4 expression
Source: Mater Today Bio. 2026 Jul 23;39:103491. doi: 10.1016/j.mtbio.2026.103491 (PMC13446093; doi:10.1016/j.mtbio.2026.103491)
Supplement: Supplementary file 1 [file mmc1.docx]

**Supplementary data**

*of*

**Self-assembled nano-PROTACs potentiate colorectal cancer immunotherapy via downregulating PD-L1 and GPX4 expression**

Xueping Luo,^a,1^ Yixin Liu,^a,1^ Guangmiao Chen,^b,1^ Xinrui Ma,^a^ Pingping Xu,^a^ Yutong Li,^a^ Longyu Zheng,^a^ Rongrong Zheng,^b^ Youqin Xu,^a,^* Shiying Li,^b,^* Linping Zhao^a,b,^*

*^a^ Key Laboratory of Biological Targeting Diagnosis, Therapy and Rehabilitation of Guangdong Higher Education Institutes, the NMPA and State Key Laboratory of Respiratory Disease, The Fifth Affiliated Hospital, Guangzhou Medical University, Guangzhou 510700, P. R. China.*

*^b^ Guangdong Provincial Key Laboratory of Molecular Target & Clinical Pharmacology, the NMPA and State Key Laboratory of Respiratory Disease, School of Pharmaceutical Sciences and the Fifth Affiliated Hospital, Guangzhou Medical University, Guangzhou 511436, P. R. China.*

* Corresponding Author Address: 2023991033@gzhmu.edu.cn; lisy-sci@gzhmu.edu.cn; zlp2022@gzhmu.edu.cn

^1^ These authors contributed equally to this work.

**Fig. S1.** TEM image of dBET57 nanoparticles. Scale bar: 100 nm.

**Fig. S2.** TEM image of TA/Fe network. Scale bar: 100 nm.

**Fig. S3.** The standard curve of dBET57 analyzed by HPLC.

**Fig. S4.** Representative western blot membrane displaying the expression of BRD4. Quantitative densitometric analysis of BRD4 proteins.

**Fig. S5.** Flow cytometry plots of CT26 cells incubated with the indicated concentrations of dBET@TF or for various time points.

**Fig. S6.** Representative micrographs and fluorescence quantitative analysis of CT26 cells after 24 h incubation with Cy5.5/dBET@TF and inhibitors targeting distinct endocytic routes. Scale bar: 20 μm.

**Fig. S7.** Quantitative fluorescence analysis of PI in CT26 cells after treatment with PBS, TA/Fe, dBET57, and dBET@TF. Statistical significance was indicated as ***P < 0.001.

**Fig. S8.** Representative western blot membrane displaying the expression of FSP1. Quantitative densitometric analysis of FSP1 proteins.

**Fig. S9.** Extracellular ATP levels of CT26 cells treated with various treatments. Statistical significance was indicated as **P <0.01 and ***P < 0.001.

**Fig. S10.** MHC-I expression on DCs following treatment with the indicated agents. Statistical significance was indicated as ***P < 0.001.

**Fig.S11.** Representative flow cytometry plots and quantification of the proportion of CD3⁺TIM3⁺ T cells following the indicated treatments. Statistical significance was indicated as *P <0.05, **P <0.01 and ***P < 0.001.

**Fig.S12.** Representative flow cytometry plots and quantification of the proportion of CD8⁺IFNγ⁺ T cells following the indicated treatments. Statistical significance was indicated as *P <0.05, **P <0.01 and ***P < 0.001.

**Fig. S13.** H&E stained sections of major organs harvested from treated mice. Scale bar: 200 μm.

**Fig. S14.** Serum levels of liver function markers (ALT and AST) and renal function indicators (UA and BUN).

**Fig. S15.** Representative tumor images and tumor volumes from the CT26 orthotopic tumor model following intravenous administration of PBS, TA/Fe, dBET57 or dBET@TF. Statistical significance was indicated as **P <0.01 and ***P < 0.001.

**Fig.S16.** (A) Tumor volumes, (B) tumor weights, and representative tumor images from the CT26 tumor rechallenge experiment in surviving mice following intravenous administration of PBS, αPD-1, TA/Fe + αPD-1, dBET57 + αPD-1, or dBET@TF + αPD-1. Statistical significance was indicated as **P <0.01 and ***P < 0.001.

**Fig.S17.** Representative flow cytometry plots and quantification of the proportion of CD8⁺CD44⁺CD62L^+^ T cells following the indicated treatments. Statistical significance was indicated as ***P < 0.001.

**Fig. S18.** The percentage of CD3^+^CD4^+^ T cells in the spleen and (B) lymph node after treatment with PBS, αPD-1, TA/Fe + αPD-1, dBET57 + αPD-1, and dBET@TF + αPD-1. Statistical significance was indicated as *P <0.05, **P <0.01 and ***P < 0.001.

**Fig. S19.** Immunofluorescent detection of CD8-positive cells in tumor tissue following various treatments. Scale bar: 100 μm.

**Fig. S20.** (A) Representative flow cytometry plots and quantification of the proportion of CD8^+^TNFα^+^ T cells following the indicated treatments. (B) Representative flow cytometry plots and quantification of the proportion of CD8^+^IFNγ^+^ T cells following the indicated treatments. Statistical significance was indicated as *P< 0.05 and ***P < 0.001.
